# miR-433 Inhibits Neuronal Growth and Promotes Autophagy in Mouse Hippocampal HT-22 Cell Line

**DOI:** 10.3389/fphar.2020.536913

**Published:** 2020-11-23

**Authors:** Chunli Xu, Qingke Bai, Chen Wang, Qiuyu Meng, Yuming Gu, Qiwei Wang, Wenjie Xu, Ying Han, Yong Qin, Song Jia, Junfang Zhang, Jie Xu, Jiao Li, Miao Chen, Feng Wang

**Affiliations:** ^1^ Department of Neurology, The Seventh People’s Hospital of Integrated Traditional Chinese and Western Medicine, Affiliated to Shanghai University of Traditional Chinese Medicine, Shanghai, China; ^2^ Department of Neurology, Pudong People’s Hospital, Shanghai, China; ^3^ School of Life Science and Technology, Tongji University, Shanghai, China; ^4^ Teaching Laboratory Center of Medicine and Life Science, Tongji University School of Medicine, Shanghai, China; ^5^ Department of Neurology, Shidong hospital, University of Shanghai for Science and Technology, Shanghai, China

**Keywords:** miR-433, HT-22 cell line, autophagy, neurodegenerative diseases, cyclin-dependent kinase 12 protein, autophagy-related genes 4a

## Abstract

**Background:** MicroRNAs (miRNAs) have an increasing functional role in some neurodegenerative diseases. Autophagy, the degradation of bulk protein in the cytoplasm, is the quality control function of protein and has a protective role in the survival of neural cells. miR-433 may play a regulatory role in neurodegenerative diseases. Many aspects underlying the mechanism of miR-433 in neural development and neurodegeneration are not clear.

**Methods:** In this study, we established stable cell lines expressing miR-433 by infecting mouse hippocampal neural cell line (HT-22) cells with rLV-miR-433 and the control rLV-miR. Pre-miR-433 expression was analyzed using polymerase chain reaction (PCR). Mature miR-433 expression was measured using quantitative PCR (qPCR). The effect of miR-433 overexpression on cell proliferation was determined using a CCK-8 assay and flow cytometry. RNA interference was used to analyze the function of Cdk12 in mediating the effect of miR-433 on cell proliferation. The effect of miR-433 overexpression on cell apoptosis was determined by flow cytometry. Autophagy-related genes Atg4a, LC3B, and Beclin-1 were determined using qPCR, Western blot, or immunofluorescence. In addition, RNA interference was used to analyze the effect of Atg4a on the induction of autophagy. TargetScan 7.2 was used to predict the target genes of miR-433, and Smad9 was determined using qPCR.

**Results:** Our results indicated that miR-433 increased the expression of Atg4a and induced autophagy by increasing the expression of LC3B-Ⅱ and Beclin-1 in an Atg4a-dependent manner. In addition, miR-433 upregulated the expression of Cdk12 and inhibited cell proliferation in a Cdk12-dependent manner and promoted apoptosis in HT-22 cells under the treatment of 10-hydroxycamptothecin.

**Conclusion:** The results of our study suggest that miR-433 may regulate neuronal growth by promoting autophagy and attenuating cell proliferation. This might be a potential therapeutic intervention in neurodegenerative diseases.

## Introduction

The pathophysiology of and therapeutic options for neurodegenerative diseases are poorly understood. The five most-studied neurodegenerative diseases are Alzheimer’s disease (AD), Parkinson’s disease (PD), amyotrophic lateral sclerosis (ALS), Huntington’s disease (HD), and spinocerebellar ataxias (SCAs) (Makeyev et al., 2007; Choi et al., 2008; Haramati et al., 2010; Tao et al., 2011; Ohsumi, 2014). All these diseases are characterized by an abnormal accumulation of proteins. Mechanisms that affect the pathogenesis of neurodegenerative diseases are many and complicated. Recent studies have indicated that microRNAs (miRNAs) play a regulatory role in neural growth and development (Makeyev et al., 2007; Choi et al., 2008). Inhibition of global miRNA biogenesis results in the occurrence of neurodegeneration (Haramati et al., 2010; Tao et al., 2011).

Autophagy is a classic way to exert protein quality control. It is a bulk degradation process that delivers intracellular proteins to lysosomes to be degraded (Ohsumi, 2014). In addition to being involved in the starvation and constitutive turnover of proteins, emerging evidence indicates that autophagy plays an important role in protecting neurons against degeneration. Komatsu et al. (2006) and Hara et al. (2006) reported that mice deficient in autophagy-related 5 (Atg5) or Atg7 protein formed cytoplasmic inclusion bodies and developed neurodegenerative symptoms. This suggests that basal autophagy is essential for the survival of neurons and may be important in the pathogenesis of neurodegenerative diseases.

Mouse miR-433 is located on chromosome 12. Insulin growth factor-1(IGF-1) treatment of rat embryonic striatal stem cells (ESSC) revealed the upregulation of miR-433 and the involvement of miR-433 in neural proliferation and differentiation (Pati et al., 2014). Wang et al. showed that fibroblast growth factor 20 (FGF20) is one target of miR-433, that polymorphism in the miR-433 binding site of FGF20 conferred risk for Parkinson’s disease, and that the expression of FGF20 is associated with α-synuclein that forms aggregates in Parkinson’s disease (Wang et al., 2008). Therefore, miR-433 may play a regulatory role in neurodegenerative disease. Many aspects underlying the mechanism of miR-433 in neural development and neurodegeneration are not clear.

In this study, we performed *in vitro* tests with mouse hippocampal HT-22 cells to explore the function of miRNA-433 in neural development. Our results indicated that miR-433 promoted neural autophagy by increasing Atg4a, LC3B, and Beclin-1, and inhibited neural cell proliferation by attenuating the cell cycle in cyclin-dependent kinase 12 protein (Cdk12)-dependent cells.

## Materials and Methods

### Construction of miR-433 Plasmid and Lentivirus Packaging

Mouse precursor miR-433 (pre-miR433, miRbase accession No. MI0001525) was synthesized, cloned into the pLVX-ZsGreen-miRNA-Puro vector (pLVX-ZsGreen-mmu-miR-433-Puro), and packaged into the lentivirus rLV-ZsGreen-mmu-miR-433-Puro (referred to as rLV-miR-433) by the Yumeibo Biotech Company (Shanghai, China). The final titers ranged from 10^7^ to 10^8 ^TU/ml.

### Cell Culture, Infection, and Monoclone Screen

The mouse hippocampal HT-22 cell line was purchased from the Xiaoying Biotech Company (Shanghai, China). HT-22 cells were maintained at 37°C and 5% CO_2_ in Dulbecco’s modified Eagle medium (DMEM)/high glucose (Gibco, Thermo Fisher Scientific, Waltham, MA, United States) supplemented with 10% fetal bovine serum (FBS) and 100 U/ml penicillin/streptomycin (Gibco). HT-22 cells were infected with rLV-miR-433 viral particles or with the control rLV-ZsGreen-Puro (referred to as rLV-miR) viral particles at a concentration of 5 × 10^6^ TU/10^6^ cells. The green fluorescent protein (GFP) expression in the vector was used to determine transfection efficiencies. After 48 h of infection, the HT-22 cells were passaged. In addition, several monoclones were selected by choosing cell clones containing GFP as seen with a fluorescent microscope. The selected clones were further cultured into stable miR-433-infected HT-22 cells (rLV-miR-433), which, along with the control-infected cells (rLV-miR), were used for all further analysis.

Small interfering RNAs (siRNAs) against the expression of Cdk12 (siCdk12), Atg4a (siAtg4a), and negative control siRNA (NC siRNA) were chemically synthesized by Sangon Biotech Co. Ltd. (Shanghai, China). The siCdk12 sequence was as follows: sense: 5′-GCA​GUC​GUC​AUU​CCA​GUA​UTT-3′, antisense: 5′-AAG​GUG​UCU​GAA​UCA​GAG​CTT-3′. The siAtg4a sequence was as follows: sense: 5′-CCU​UGU​UCA​GAA​GGA​AAU​UTT-3′, antisense: 5′-AAU​UUC​CUU​CUG​AAC​AAG​GTT-3′. The NC siRNA sequence was as follows: sense: 5′-GUGAGCGUCUAUAUACCAUdTdT-3′, antisense: 5′-AUGGUAUAUAGACGCUCACdTdT-3′. Cells were placed into 6-well cell culture plates and cultured to 60–70% confluence. siRNAs (100 pmol/well) were transfected into stable rLV-miR and rLV-miR-433 cells using Lipofectamine 2000 (Invitrogen; Thermo Fisher Scientific) according to the protocols of the manufacturer. After 6 h of transfection, the culture medium in each well was replaced with fresh complete medium.

### MicroRNAs Extraction, Quantitative Polymerase Chain Reaction, and Regular Polymerase Chain Reaction

The kits for miRNA isolation (DP501), miRNA first-strand cDNA synthesis (KR211), and miRNA quantitative polymerase chain reaction (qPCR) detection (FP411) were purchased from TIANGEN Biotech Co. Ltd. (Beijing, China). Briefly, the miRNA isolation procedure was as follows: Cells were lysed in lysis buffer and kept at 25°C for 5 min. After adding 200 μl of chloroform, the mixture was vortexed for 15 s, incubated at room temperature for 5 min, and centrifuged at 4°C and 10,000 g for 15 min. The upper layer of the water phage was transferred to a new tube to which the proper volume of ethanol was added. The mixture was then vortexed, transferred to a spin column, and centrifuged to obtain the eluate, to which the proper volume of ethanol was added. The mixture was vortexed, transferred to a spin column, and centrifuged at 10,000 g for 30 s at room temperature. After rinsing twice, the miRNA in the spin column was dissolved in RNase-free ddH_2_O.

For miRNA first-strand cDNA synthesis, up to 2 μg of miRNA was mixed with reaction buffer and enzymes for a final volume of 20 μl for each test. The mixtures were treated at 42°C for 60 min and then at 95°C for 5 min to denature the enzymes.

For miRNA qPCR, we used the StepOnePlus^™^ Real-Time PCR System (Thermo Fisher Scientific). The reaction system (20 μl) contained 10 μl of 2× miRNA Premix (with SYBR^®^ and ROX^™^ calibrated dyes), forward primers (synthesized by Sangon Biotech) and reverse primers (provided by the kit, 10 μm for each), and 2 μl of miRNA first-strand cDNA. qPCR reactions were performed in triplicate and the data were normalized against the levels of U6 RNA. The sequences of mature mmu-miR-433-5p (MIMAT0001419) and mmu-miR-433-3p (MIMAT0001420) were used as forward primers in the qPCR analysis. The forward primer sequence of mmu-miR-433-5p was 5′-TAC​GGT​GAG​CCT​GTC​ATT​ATT​C-3′ and that of mmu-miR-433-3p was 5′-ATC​ATG​ATG​GGC​TCC​TCG​GTG​T-3′. Regular PCR was performed to detect mouse pre-miR-433 expression. The forward primer for pre-miR-433 was 5′-TGC​CCG​GGG​AGA​AGT​ACG​GT-3′ and the reverse primer was 5′-GGG​CTG​CCT​TCA​TGG​TGT​GG-3′. The expression of pre-miR-433 was analyzed after 38 cycles of regular PCR.

### Analysis of Cell Viability

Cell viability was assessed using the CCK-8 assay (Lot 8620; Signalway Antibody, College Park, MD, United States). HT-22 cells infected with rLV-miR-433 and rLV-miR were cultured in 96-well plates (5 × 10^3^ cells/well). After incubation for 0–4 days, the cells were incubated with CCK-8 (10 μl of CCK-8 added to each well) for 1 h at 37°C. Absorbance was measured at 450 nm using a SpectraMax M3 microplate reader (Molecular Devices, San Jose, CA, United States). In addition, the cells transfected with siCdk12 for 36 h were then cultured in 96-well plates (1 × 10^4^ cells per well). After incubation for 24 and 48 h, cell viability was determined using the CCK-8 assay as described above.

### Cell Cycle Analysis

We purchased the cell cycle analysis kit from Beyotime (Lot C1052, Shanghai, China). Cells were cultured in 6-well plates, digested, collected, washed with cold phosphate buffered saline (PBS), fixed in iced 70% ethanol at 4°C for 3 h, washed again with cold PBS, and stained with propidium iodide (PI)/RNase A mixture at 37°C for 30 min in the dark. Finally, cells were analyzed using the Fluorescence-Activated Cell Sorting (FACS) Calibur system (FACSVerse^™^, BD Biosciences, San Jose, CA, United States).

### RNA Extraction, Real-Time Polymerase Chain Reaction, and Quantitative Polymerase Chain Reaction

Total RNA was purified with RNAiso Plus (D9108B, Takara Bio USA, Mountain View, CA, United States). Reverse transcription (RT) was performed using 5× PrimeScript RT Master Mix (Takara Bio USA) at 37°C for 15 min and at 85°C for 5 s to denature enzymes. cDNA samples were analyzed using qPCR (SuperReal SYBR Green PreMix Plus, FP205, TIANGEN Biotech) with the appropriate primers. For qPCR, 10 ng of RNA was used for each test and reactions were performed in triplicate on a StepOnePlus thermocycler (Thermo Fisher Scientific). The data were normalized against the levels of β-actin mRNA. The primers used were as follows: Cdk12, forward: 5′-AAC​AGC​TAA​TGG​AAG​GAC​TGG-3′ and reverse: 5′-CAG​AGT​TAT​AGA​GCC​GAG​CAA​G-3′; Atg4a, forward: 5′-ATT​CCT​TGG​CTG​TTT​ATG​TTT​CC-3′ and reverse: 5′-GTG​TCT​CTA​CTC​TGA​TTG​GAT​GC-3′; Smad9, forward: 5′-CTG​ACC​AAG​ATG​TGT​ACT​ATC​CG-3′ and reverse: 5′-TCA​GAA​CTT​TGT​CCA​GCC​AC-3′; and β-actin, forward: 5′-GAG​GTA​TCC​TGA​CCC​TGA​AGT​A-3′ and reverse: 5′-GCT​CGA​AGT​CTA​GAG​CAA​CAT​AG-3′.

### Cell Apoptosis Analysis

Cell apoptosis was determined with PI staining using the same cell cycle analysis kit from Beyotime. 10-Hydroxycamptothecin (HCPT) was purchased from Sangon Biotech. Briefly, HT-22 cells infected with rLV-miR-433 and rLV-miR were placed in 6-well plates. The cells were then digested, collected, washed with cold PBS, and stained in PI/RNase A mixture at 37°C for 30 min in the dark. Finally, cells were analyzed using a FACS Calibur system (FACSVerse^™^).

### Cell Autophagy Detection

In this study, autophagy was induced by starving the HT-22 cells by culturing them in a medium with 0.05% FBS for different periods. Next, autophagy in the HT-22 cells was detected by visualizing LC3B puncta using fluorescence microscopy. Adenovirus-expressing mCherry-GFP-LC3B fusion protein (Ad-mCherry-GFP-LC3B) was purchased from Beyotime (Lot C3011). The protocol was based on that suggested by the manufacturer. Briefly, HT-22 cells infected with rLV-miR-433 and rLV-miR were prepared at the proper density in 6-well plates on the day of the experiment. Cells were then infected with Ad-mCherry-GFP-LC3B at 10 MOI (multiplicity of infection) for each well containing 5 × 10^5^ cells. Twenty-four hours after infection, the growth medium was aspirated and replaced with fresh growth medium and the cells were cultured for another 24 h. At the end of the treatment, cells were visualized using an EVOS microscope (Life Technologies). The number of cells with LC3B puncta per field was counted.

### Western Blotting

Whole-cell proteins were extracted using RIPA lysis buffer [P0013B, Beyotime, 50 mM Tris (pH 7.4), 150 mM NaCl, 1% Triton X-100, 1% sodium deoxycholate, 0.1% sodium dodecyl sulfate (SDS), sodium orthovanadate, sodium fluoride, ethylenediaminetetraacetic acid (EDTA), and leupeptin]. For electrophoresis, 20 μg of each protein extract was loaded onto denatured 12% polyacrylamide gel and then transferred to a polyvinylidene difluoride (PVDF) membrane (Millipore Sigma, Burlington, MA, United States). The membrane was incubated in 5% skim milk for 1 h at 25°C followed by overnight incubation at 4°C with the primary antibodies of LC3B (sc-398822, Santa Cruz Biotechnology, Santa Cruz, CA, United States), Beclin-1 (11306-1-AP, Proteintech, Rosemont, IL, United States), Atg4a (ab108322, Abcam, Cambridge, United Kingdom), and β-actin (60008-1-lg, Proteintech). The next day, the membrane was incubated at 25°C for 1 h with anti-mouse secondary antibody conjugated with horseradish peroxidase antibodies (HRP, SA00001-1, Proteintech) or with anti-rabbit secondary antibody conjugated with HRP (SA00001-2, Proteintech). HRP signals were detected using the enhanced chemical luminol reagent (Proteintech) on an Amersham Imager 600 (GE Healthcare Bio-Sciences Corp., Piscataway, NJ, United States).

### Statistical Analysis

Statistical significance was determined using the Student *t* test with GraphPad Prism 7 (GraphPad Software, San Diego, CA, United States). For cell cycle and apoptosis, data analysis was performed using FlowJo ver. 10 software (BD Biosciences). For autophagy detection, cells were quantified using ImageJ_ver. 1.8.0 software (NIH, Bethesda, MD, United States). A minimum of five randomly chosen fields were quantified for the assays. Western blot analysis was performed using ImageJ_ver. 1.8.0 software. In all cases, *p* < 0.05 was considered statistically significant.

## Results

### Generation of a Lentivirus Expressing miR-433

**FIGURE 1:**
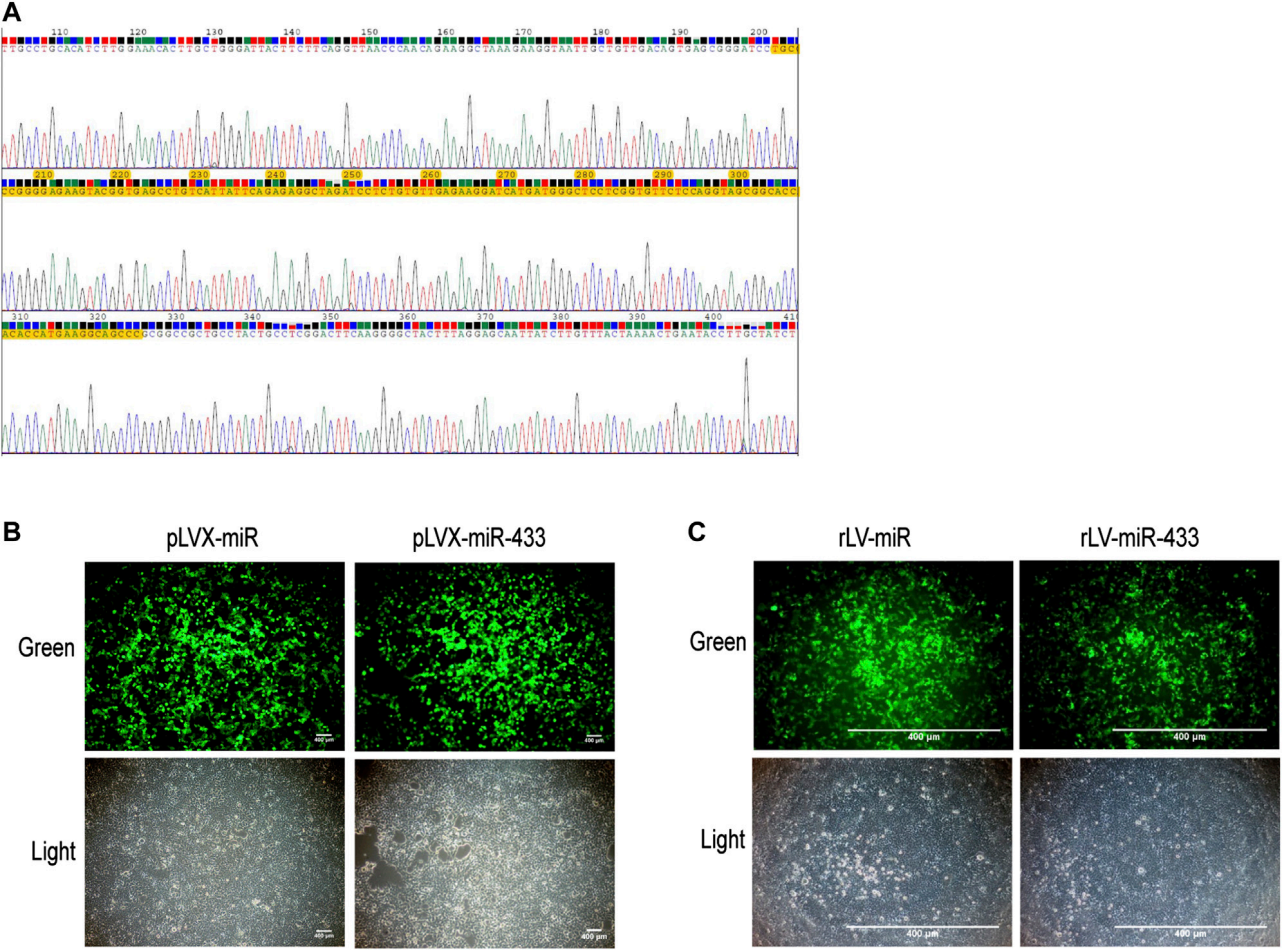
Construction of pLVX-ZsGreen-mmu-miR-433-Puro plasmid and rLV-ZsGreen-mmu-miR-433-Puro (rLV-miR-433) virus. **(A)** The sequencing of pLVX-ZsGreen-mmu-miR-433-Puro. The yellow highlighted area is the correct mmu pre-miR-433 sequence (124 bp). **(B)** Transfection of HEK293 cells using pLVX-ZsGreen-mmu-miR-433-Puro. Top row: observation under green fluorescent light; bottom row: observation under white light. Scale bar = 400 µm. **(C)** Infection of HEK293 cells with rLV-miR-433 lentivirus. Top row: observation under green fluorescent light; bottom row: observation under white light. Scale bar = 400 µm.

To observe the effects of miR-433, we first generated a lentivirus that expressed miR-433. We selected the sequence of mouse step-loop pre-miR433 and constructed the recombinant plasmid pLVX-ZsGreen-mmu-miR-433-Puro, in which the inserted sequence was correct (Figure 1A). HEK293 cells were transfected with pLVX-ZsGreen-mmu-miR-433-Puro and expressed GFP (Figure 1B). Next, we prepared a lentivirus (rLV-mir-433) using pLVX-ZsGreen-mmu-miR-433-Puro and obtained a high titer. Finally, HEK293 cells were infected with rLV-mir-433, and GFP was clearly expressed (Figure 1C). Collectively, these observations suggested that a lentivirus expressing pre-miR-433 had been successfully prepared.

### Establishment of Stable HT-22 Cell Lines Overexpressing miR-433

**FIGURE 2:**
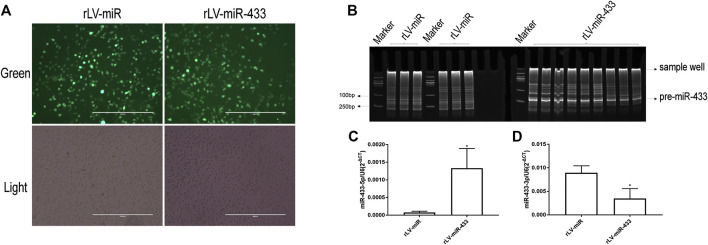
Identification of overexpression of mmu miR-433 in HT-22 cells. **(A)** Selected monoclonal HT-22 cells infected with rLV-miR or rLV-miR-433 virus particles. Top row: observation under green fluorescent light; bottom row: observation under white light. Scale bar = 400 µm. **(B)** General PCR analysis of pre-miR-433 expression in monoclonal HT-22 cells infected with rLV-miR or rLV-miR-433. Products were obtained using polyacrylamide gel electrophoresis (PAGE). Arrows indicate the mmu pre-miR-433 band (124 bp), sample wells (250 bp), and marker bands (100 bp). **(C)** qPCR analysis of mmu mature miR-433 expression in selected monoclonal HT-22 cells infected with rLV-miR or rLV-miR-433. Histogram shows the mmu mature miR-433-5p expression (MIMAT0001419) (*n* = 3). **(D)** Histogram shows the mmu mature miR-433-3p expression (MIMAT0001420) (*n* = 3). Relative expression was calculated using the relative cycle threshold quantification method (2^−ΔCt^) and normalized to U6. Significance was determined by comparing rLV-miR-433 to rLV-miR (control) using the Student *t* test. Error bars represent standard deviation (SD). *p < 0.05; **p < 0.01.

To elucidate the effect of miR-433 on neural cells, we established stable HT-22 cell lines that overexpressed miR-433. First, HT-22 cells were infected with rLV-miR-433 and the control rLV-miR virus particles. Then, positive monoclonal cells expressing GFP were selected using a microscope and were continued to be cultured (Figure 2A). Second, we extracted total RNA from the two established stable cell lines in which pre-miR-433 and mature miR-433 were detected, respectively. Figure 2B shows that pre-miR-433 expression was significantly higher in the rLV-miR433 group than in the rLV-miR group. Moreover, qPCR results showed that the level of mature miR-433-5p was significantly higher in the rLV-miR433 group than in the rLV-miR group (0.00133 ± 0.00056 vs. 0.00008 ± 0.00003, *p* = 0.0317) (Figure 2C). However, the level of mature miR-433-3p was significantly lower in the rLV-miR433 group than in the rLV-miR group (0.00348 ± 0.00213 vs. 0.00893 ± 0.00150, *p* = 0.0065) (Figure 2D). For mature miR-433, it is possible that only miR-433-5p had a function in the rLV-miR433 group. Together, these results suggested that we successfully established stable HT-22 cell lines that overexpressed miR-433.

### miR-433 Inhibits the Growth of HT-22 Cells via Cdk12

**FIGURE 3:**
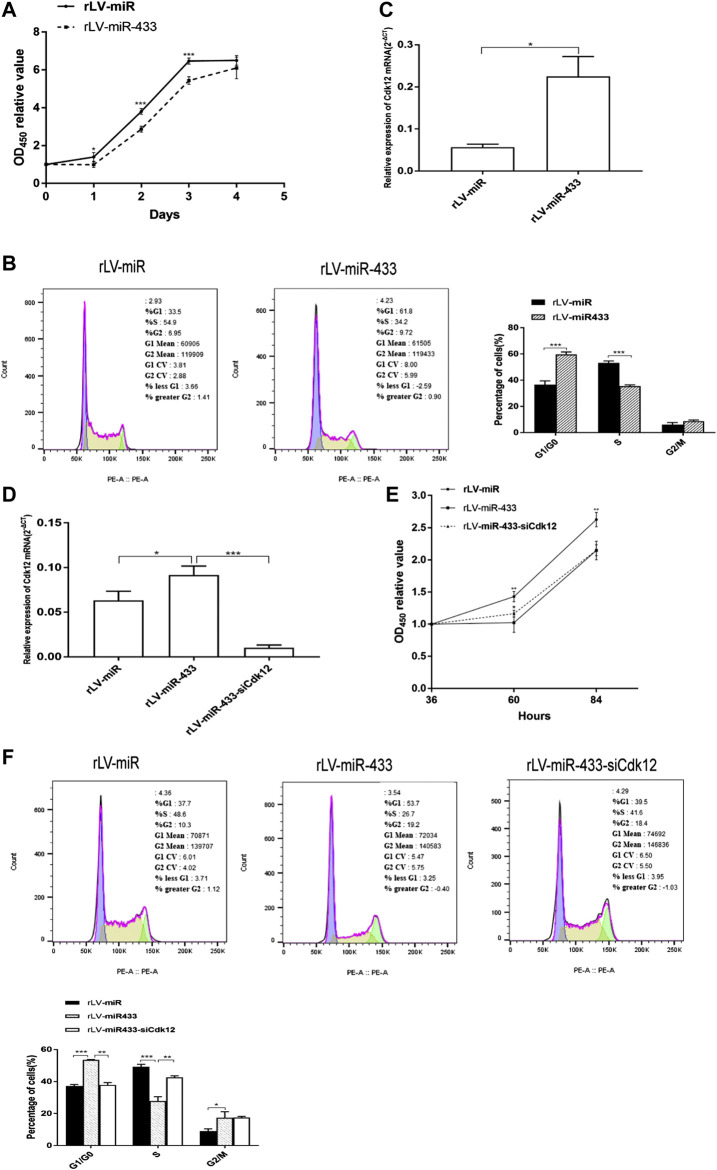
Cell proliferation and cell cycle distribution in the rLV-miR, rLV-miR-433, and rLV-miR-433-siCdk12 groups. Selected monoclones of the rLV-miR and rLV-miR-433 groups were thawed at the same time and further cultured to analyze mRNA expression and cell cycle distribution after 72 h of thawing. **(A)** Cell proliferation assay using CCK-8 in the rLV-miR and rLV-miR-433 groups. Data are given as mean ± standard deviation (SD) (*n* = 4). Error bars represent SD. *p < 0.05; ***p < 0.001. **(B)** Cell cycle distribution in HT-22 cells with separate rLV-miR and rLV-miR-433 overexpression. Left and middle: representative image of the cell cycle; right: histogram. Data are given as mean ± SD (*n* = 3). Error bars represent SD. ***p < 0.001. **(C)** qPCR was used to detect Cdk12 in HT-22 cells from the rLV-miR and rLV-miR-433 groups. **(D)** qPCR was used to detect Cdk12 in HT-22 cells from the rLV-miR, rLV-miR-433, and rLV-miR-433-siCdk12 groups. β-Actin was used as an internal control. Data are given as mean ± SD (*n* = 3). Error bars represent SD. *p < 0.05; ***p < 0.001. **(E)** Cell proliferation assay using CCK-8 in the rLV-miR, rLV-miR-433, and rLV-miR-433-siCdk12 groups. Data are given as mean ± SD (*n* = 4). Error bars represent SD. *p < 0.05; **p < 0.01. **(F)** Cell cycle distribution in the rLV-miR, rLV-miR-433, and rLV-miR-433-siCdk12 groups. Top: representative image of the cell cycle; bottom: histogram. Data are given as mean ± SD (*n* = 3). Error bars represent SD. *p < 0.05; **p < 0.01; ***p < 0.001.

To validate the effect of miR-433 on the growth of HT-22 cells, cell proliferation of the two stable HT-22 cell lines, the rLV-miR group and the rLV-miR-433 group, was measured using the CCK-8 assay. Figure 3A shows that the HT-22 cells grew significantly slower in the rLV-miR-433 group than in the rLV-miR group at days 1–3 (0.9931 vs. 1.3999, *p* = 0.0282; 2.8672 vs. 3.8064, *p* = 0.0001; 5.4355 vs. 6.4686, *p* = 0.0002, respectively). Furthermore, the cell cycle was detected in the two cell lines. Figure 3B shows that the percentage of cells in the S stage was significantly lower in the rLV-miR-433 group than in the rLV-miR group (35.40 ± 1.03% vs. 53.30 ± 1.39%, *p* = 0.000098). However, the percentage of cells in the G0/G1 stage was significantly higher in the rLV-miR-433 group than in the rLV-miR group (59.73 ± 1.84% vs. 36.63 ± 2.83%, *p* = 0.000664) and the percentage of cells in the G2/M stage was not significantly different between the groups (8.82 ± 0.82% vs. 6.09 ± 1.59%, *p* = 0.078). In particular, qPCR analysis indicated that the expression of Cdk12 was significantly upregulated in the rLV-miR-433 group compared to that in the rLV-miR group (0.2249 ± 0.0472 vs. 0.0569 ± 0.0070, *p* = 0.0288) (Figure 3C).

To identify whether Cdk12 affects the growth of the miR-433 group, we analyzed cell proliferation in the rLV-miR, rLV-miR-433, and rLV-miR-433-siCdk12 (Cdk12 knockdown) groups. First, qPCR analysis presented in Figure 3D shows that Cdk12 expression was significantly higher in the rLV-miR-433 group than in the rLV-miR group (0.0915 ± 0.0101 vs. 0.0634 ± 0.0102, *p* = 0.0272) and lower in the rLV-miR-433-siCdk12 group than in the rLV-miR-433 group (0.0102 ± 0.0031 vs. 0.0915 ± 0.0101, *p* = 0.0002). Second, Figure 3E shows that proliferation of HT-22 cells was lower in the rLV-miR-433 group than in the rLV-miR group at 60 and 84 h (1.0234 vs. 1.4297, *p* = 0.0061 and 2.1454 vs. 2.6262, *p* = 0.0023, respectively). However, proliferation of HT-22 cells had increased faster in the rLV-miR-433-siCdk12 group than in the rLV-miR-433 group at 60 h (1.1627 vs. 1.0234, *p* = 0.0434) but was not significantly different in the rLV-miR-433-siCdk12 group and the rLV-miR-433 group at 84 h (2.1489 vs. 2.1454, *p* = 0.9680). The siCdk12 effect and the reduction of Cdk12 by siCdk12 within 60 h after transfection allowed the rLV-miR-433-siCdk12 group to grow faster than the rLV-miR-433 group. A longer post-transfection time of 84 h usually causes siCdk12 to lose its interference effect; therefore, the growth rate of the rLV-miR-433-siCdk12 group had decreased to the same rate as that of the rLV-miR-433 group at 84 h. Third, as seen in Figure 3F, the percentage of cells in the S stage was significantly lower in the rLV-miR-433 group than in the rLV-miR group (27.90 ± 2.62% vs. 49.30 ± 1.48%, *p* = 0.00089), whereas the percentage of cells in the G0/G1 stage was significantly higher (53.50 ± 0.35% vs. 37.17 ± 1.01%, *p* = 0.0004) and that in the G2/M stage was also higher (17.43 ± 3.68% vs. 9.07 ± 1.38%, *p* = 0.0453) in the rLV-miR-433 group than in the rLV-miR group. However, the percentage of cells in the S stage was significantly higher in the rLV-miR-433-siCdk12 group than in the rLV-miR-433 group (42.63 ± 1.00% vs. 27.90 ± 2.62%, *p* = 0.0050), whereas the percentage of cells in the G0/G1 stage was significantly lower in the rLV-miR-433-siCdk12 group than in the rLV-miR-433 group (37.93 ± 1.46% vs. 53.50 ± 0.35%, *p* = 0.0019). The percentage of cells in the G2/M stage did not change significantly in the rLV-miR-433-siCdk12 group compared with that in the rLV-miR-433 group (17.53 ± 0.75% vs. 17.43 ± 3.68%, *p* = 0.9671). These results showed that miR-433 may attenuate the growth of HT-22 cells by altering the cell cycle distribution that may be dependent on Cdk12.

### miR-433 Increases the Apoptosis of HT-22 Cells

**FIGURE 4:**
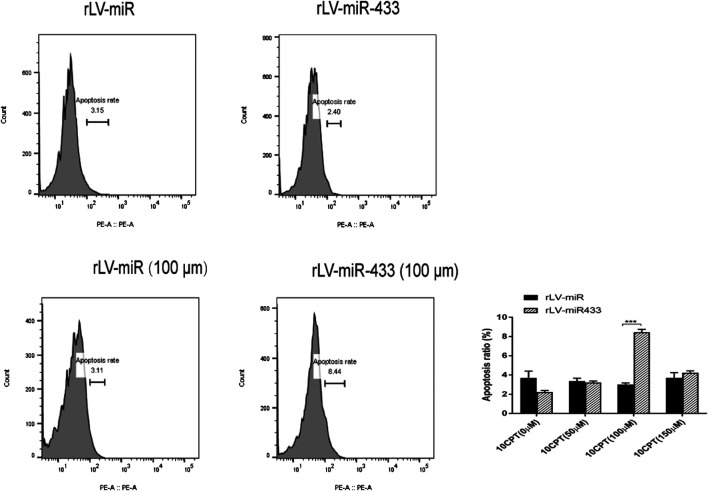
Apoptosis was detected in the rLV-miR and rLV-miR-433 groups. Upper left and middle: Representative images of cell apoptosis assay without treatment with HCPT. Lower left and middle: Representative images of cell apoptosis assay after treatment with HCPT (100 µM) for 4 h. All samples were measured using flow cytometry with PI staining. Significance was determined by comparing the apoptosis in the rLV-miR-433 group to that of the rLV-miR group using the Student *t* test. All data are given as mean ± SD (*n* = 3). ***p < 0.001.

To further investigate whether changes in cell growth and the cell cycle are associated with apoptosis, we treated rLV-miR- and rLV-miR-433-infected HT-22 cells with HCPT to induce apoptosis. HCPT is a specific topoisomerase Ⅰ inhibitor that can inhibit multiple cancers by decreasing cell growth and inducing cell apoptosis (Song et al., 2019). Our results showed that there was no difference between the apoptosis rates of the rLV-miR-433 and rLV-miR groups not treated with HCPT (Figure 4). After treatment with 100 µm of HCPT, the apoptosis rate was significantly higher in the rLV-miR-433 group than in the rLV-miR group (8.43 ± 0.3202 vs. 3.00 ± 0.1735, *p* = 0.0001). However, there was no significant difference between the apoptosis rates after treatment with 50 and 150 µm of HCPT. These data suggested that miR-433 promoted the apoptosis of HT-22 cells.

### miR-433 Promotes Atg4a-Related Autophagy in HT-22 Cells

**FIGURE 5:**
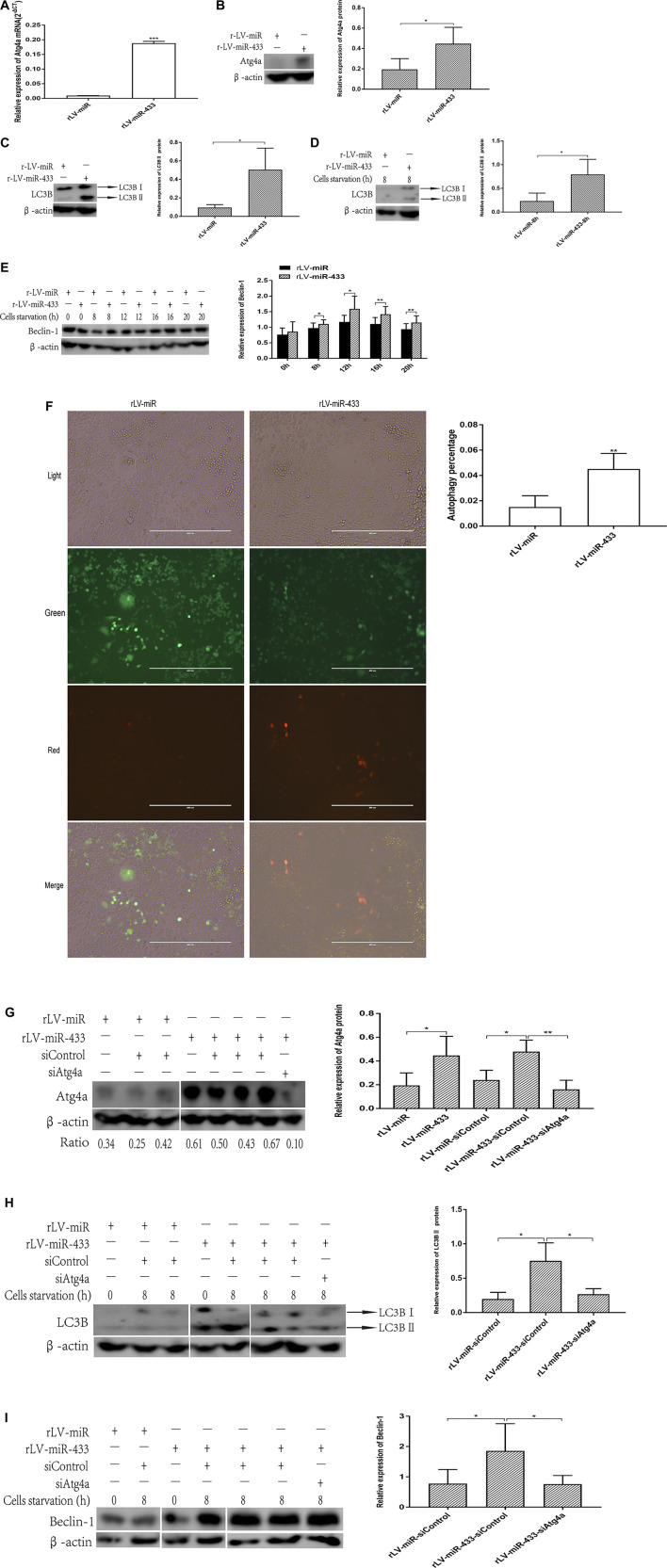
miR-433 promotes autophagy dependent on Atg4a. **(A)** qPCR was used to detect Atg4a in HT-22 cells from the rLV-miR group and the rLV-miR-433 group. **(B)** Western blotting using anti-Atg4a was used to detect Atg4a expression in HT-22 cells from the rLV-miR group and the rLV-miR-433 group. Left: image of bands; right: histogram. **(C)** Western blotting using anti-LC3B was used to detect LC3B expression in HT-22 cells from the rLV-miR group and the rLV-miR-433 group. Left: image of bands (upper arrow indicates LC3B-Ⅰ and lower arrow indicates LC3B-Ⅱ); right: histogram shows the relative expression of LC3B-Ⅱ. **(D)** Western blotting using anti-LC3B was used to detect LC3B expression in HT-22 cells from the rLV-miR group and the rLV-miR-433 group under starvation for 8 h. Left: image of bands (upper arrow indicates LC3B-Ⅰ and lower arrow indicates LC3B-Ⅱ); right: histogram to show the relative expression of LC3B-Ⅱ. **(E)** Western blotting using anti-Beclin-1 was used to detect Beclin-1 expression in HT-22 cells from the rLV-miR group and the rLV-miR-433 group under starvation for 0–20 h. Left: image of bands; right: histogram. **(F)** Autophagy detection in the rLV-miR and rLV-miR-433 groups via infection with the Ad-mCherry-GFP-LC3B virus. Arrows indicate red positive cells with autophagosomes. Red dots represent cells containing LC3B protein binding with autophagosomes, which are identified as autophagy cells. Significance was determined by comparing the red positive cells of the rLV-miR-433 group to those of the rLV-miR group (*n* = 5). Error bars represent SD; **p < 0.01. Five visual fields of each cell group were counted, and the positive percentage of cells was calculated using the Student *t* test. Scale bar = 400 µm. **(G)** Western blotting using anti-Atg4a was used to detect Atg4a expression in HT-22 cells from the rLV-miR, rLV-miR-433, and rLV-miR-433-siAtg4a groups. β-Actin was used as the internal control. Left: image of bands; right: histogram; ratio: band density ratio of each group to β-actin. **(H)** Western blotting using anti-LC3B was used to detect LC3B expression in HT-22 cells from the rLV-miR, rLV-miR-433, and rLV-miR-433-siAtg4a groups at the beginning of induction of starvation (0 h) and under starvation for 8 h. Left: image of bands (upper arrow indicates LC3B-Ⅰ and lower arrow indicates LC3B-Ⅱ); right: histogram shows the relative expression of LC3B-Ⅱ. **(I)** Western blotting using anti-Beclin-1 was used to detect Beclin-1 expression in HT-22 cells from the rLV-miR, rLV-miR-433, and rLV-miR-433-siAtg4a groups at the beginning of the induction of starvation (0 h) and under starvation for 8 h. Left: image of bands; right: histogram. β-Actin was used as the internal control. Data are given as mean ± SD (*n* = 3). Error bars represent SD. *p < 0.05; **p < 0.01; ***p < 0.001.

Given that miR-433 affects the growth of HT-22 cells, we wanted to see whether the change in cell growth was related to cell autophagy. First, we detected Atg4a expression in the rLV-miR and rLV-miR-433 groups. The transcription of the autophagy-related gene Atg4a was significantly higher in the rLV-miR-433 group than in the rLV-miR group (0.1882 ± 0.0065 vs. 0.0097 ± 0.0003, *p* = 0.0004) (Figure 5A). The translation of Atg4a was also significantly higher in the rLV-miR-433 group than in the rLV-miR group (0.4467 ± 0.1607 vs. 0.1949 ± 0.1044, *p* = 0.0426) (Figure 5B). Second, HT-22 cells underwent starvation to induce autophagy by culturing them in a medium that contained 0.05% FBS. LC3B-Ⅰ, LC3B-Ⅱ, and Beclin-1 were detected. The relative expression of LC3B-Ⅱ (activated form of LC3B) was significantly higher in the rLV-miR-433 group than in the rLV-miR group at the beginning and after 8 h of starvation [0.5036 ± 0.2342 vs. 0.0946 ± 0.0321, *p* = 0.0433 (Figure 5C) and 0.7922 ± 0.3192 vs. 0.2309 ± 0.1684, *p* = 0.0390 (Figure 5D), respectively]. Furthermore, as seen in Figure 5E, the expression of the autophagy marker Beclin-1 was higher in the rLV-miR-433 group than in the rLV-miR group after 8, 12, 16, and 20 h of starvation (8 h: 1.1058 ± 0.1321 vs. 0.9755 ± 0.1606, *p* = 0.0196; 12 h: 1.5913 ± 0.4085 vs. 1.1689 ± 0.2128, *p* = 0.0359; 16 h: 1.4169 ± 0.2490 vs. 1.1046 ± 0.2060, *p* = 0.0085; 20 h: 1.1494 ± 0.2145 vs. 0.9381 ± 0.1774, *p* = 0.0079). Notably, autophagy was significantly higher in the rLV-miR-433 group than in the rLV-miR group (0.0451 ± 0.0123 vs. 0.0150 ± 0.0089, *p* = 0.0022) (Figure 5F).

To further study whether miR-433 promoted autophagy via Atg4a, we performed the same experiments in the rLV-miR, rLV-miR-433, and rLV-miR-433-siAtg4a (Atg4a knockdown) groups. Figure 5G shows that the level of translation of Atg4a was significantly higher in the rLV-miR-433-siControl group than in the rLV-miR-siControl group (0.4781 ± 0.0977 vs. 0.2398 ± 0.0822, *p* = 0.0203). However, it was significantly lower in the rLV-miR-433-siAtg4a group than in the rLV-miR-433 group (0.1608 ± 0.0764 vs. 0.4781 ± 0.0977, *p* = 0.0064). Next, we detected the expression of LC3B-Ⅱ and Beclin-1 in these cell groups. The relative expression of LC3B-Ⅱ was higher in the rLV-miR-433 group than in the rLV-miR group 8 h after starvation induction (0.7532 ± 0.2623 vs. 0.1966 ± 0.0972, *p* = 0.0281) (Figure 5H). However, it was lower in the rLV-miR-433-siAtg4a group than in the rLV-miR-433 group (0.2698 ± 0.0776 vs. 0.7532 ± 0.2623, *p* = 0.0377). The expression of Beclin-1 was significantly higher in the rLV-miR-433-siControl group than in the rLV-miR-siControl group 8 h after starvation induction (1.8551 ± 0.8999 vs. 0.7760 ± 0.4687, *p* = 0.0360) but was lower in the rLV-miR-433-siAtg4a group than in the rLV-miR-433 group (0.7635 ± 0.2819 vs. 1.8551 ± 0.8999, *p* = 0.0483) (Figure 5I).

In addition, Smad9 (neuroprotection protein) was significantly higher in the rLV-miR-433 group than in the rLV-miR group (0.1695 ± 0.0545 vs. 0.0253 ± 0.0186, *p* = 0.0363) (Supplementary Figure S1). Collectively, these results suggested that miR-433 promotes autophagy by enhancing the expression of LC3B and Beclin-1 via mediation by Atg4a. This enhanced autophagy may increase Smad9.

## Discussion

**FIGURE 6:**
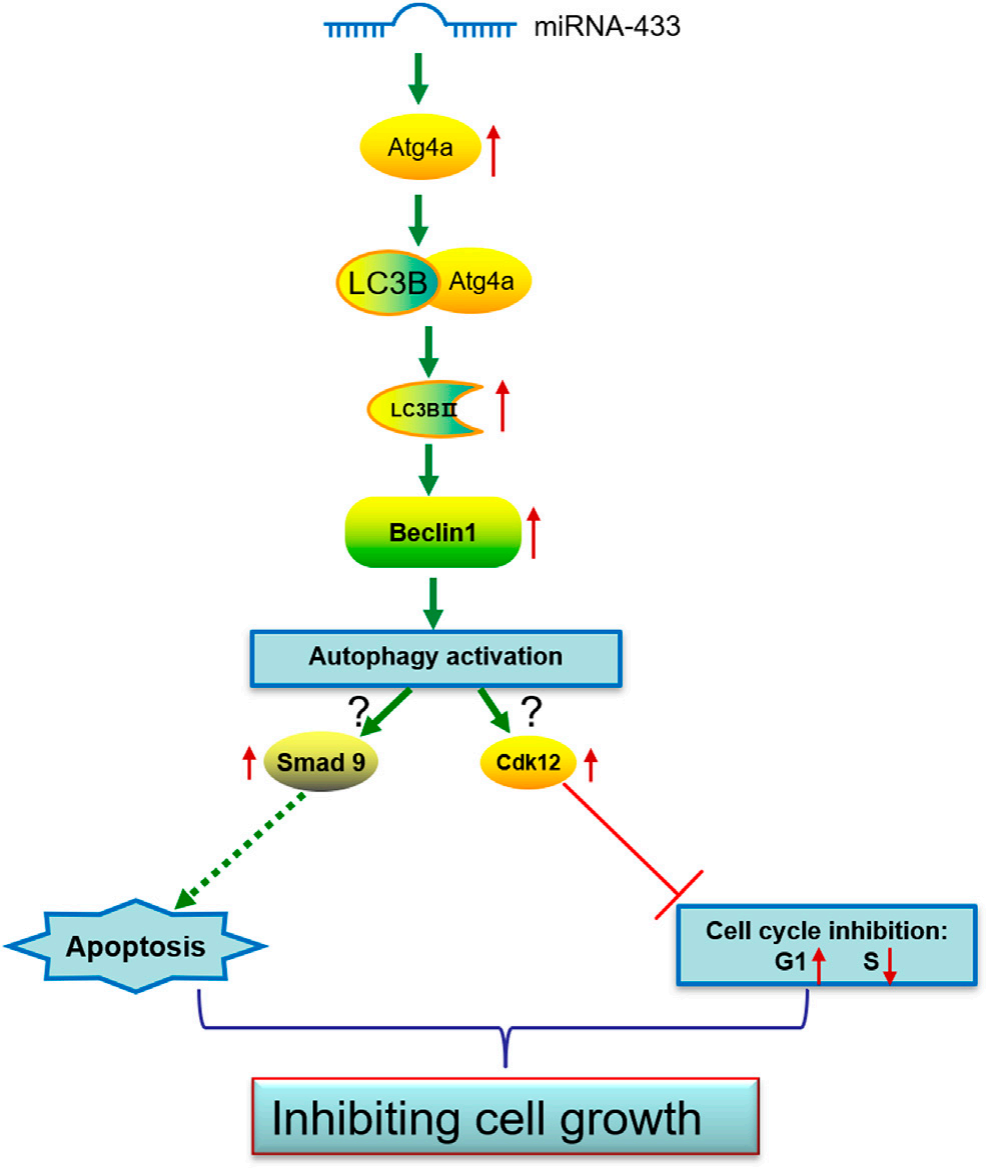
A model of proposed miR-433-induced effects on neuronal cell autophagy activation, cell cycle inhibition, and apoptosis promotion.

Autophagy has been identified to have multiple functions, including protecting neurons. To date, miRNA profiling comparisons and studies on individual miRNAs have shown that specific miRNAs are expressed in the brain and each miRNA may have a specific function in brain integrity (Sempere et al., 2004; Landgraf et al., 2007; Bak et al., 2008). Several studies have found that miR-433 can inhibit the proliferation and metastasis of some carcinomas (Shi et al., 2018; Yan et al., 2018). In this study, we demonstrated that miR-433 inhibits the proliferation of HT-22 cells by altering the cell cycle and increasing apoptosis. This inhibition of HT-22 cell proliferation may be associated with Atg4a-related autophagy (Figure 6). Our results suggest that miR-433 functions via autophagy, which may be a potential therapeutic in neurodegenerative diseases.

In this study, we found that the effect of miR-433 on cell proliferation was related to a change in the cell cycle and apoptosis. Our data indicated that miR-433 inhibits the growth of HT-22 neuronal cells. This inhibition may be mainly due to the increasing percentage of the G1 stage and the decreasing percentage of the S stage. Significantly, we found that miR-433 enhanced the expression of Cdk12 in HT-22 cells but that this effect of miR-433 was attenuated by the knockdown of Cdk12 in miR-433-overexpressed HT-22 cells. Cdk12 is a transcription-associated cyclin-dependent kinase that is essential for many cellular processes, including splicing, differentiation, and double-strand break repair (DDR) (Dubbury et al., 2018). Juan et al. (2016) reported that Cdk12 was able to maintain the genomic stability and pluripotency of embryonic stem cells. Anil reported that Cdk12 promoted G1/S progression in the human colon cancer HCT116 cell line (Manavalan et al., 2019). However, another study showed that Cdk12 had a dual role in carcinogenesis as both a tumor suppressor and an oncogene (Paculová and Kohoutek, 2017). We postulated that Cdk12 may negatively regulate the cell cycle in neuronal cells. Our findings suggest that Cdk12 determines the function of miR-433 in the cell cycle, which inhibits cell proliferation. Our results also indicate that miR-433 increases the apoptosis of HT-22 cells induced by HCPT. We believe that miR-433 inhibits neuronal cell growth by triggering apoptosis. Studies have shown that neurodegeneration is a chronic process that causes neuronal apoptosis and further loss of neurons (Giaime et al., 2017). Given that miR-433 inhibits cell proliferation and promotes apoptosis, targeting miR-433 in terminally differentiated neurons that do not proliferate may protect neural cells from damage and death by preventing apoptosis during neurodegenerative pathological processes. In addition, because of the multiple functions of autophagy, targeting miR-433 may also confer a protective role in neurons.

Autophagy is a cellular protein clearance mechanism in which portions of the cytoplasm are engulfed in double-membrane structures called autophagosomes. The autophagosomes then fuse with lysosomes, resulting in the degradation of the content of the autophagosomes. Autophagy-related genes (Atg) are required for initiating and completing the autophagy process (Levine and Klionsky, 2004). Our study found that miR-433 promoted autophagy and that Atg4a was enhanced significantly in HT-22 cells. Agt4a cysteine proteases are implicated in the formation, elongation, and fusion of the autophagic vesicles with lysosomes (Fernandez and Lopez-Otin, 2015), which suggests that miR-433 may promote autophagy via Atg4a. Furthermore, miR-433 promotes the transformation of LC3B-Ⅱ and the expression of Beclin-1. The microtubule-associated protein 1-light chain 3 (MAP1-LC3) subfamily LC3B and Beclin-1 are downstream molecules of Atg4 and are accepted markers for assessing autophagy activitly (Schaaf et al., 2016). Our results suggest that miR-433 can regulate the expression of LC3B and Beclin-1. In particular, the transformation of LC3B-Ⅱ and the expression of Beclin-1 were reduced after the knockdown of Agt4a, which can be supported by the study of Alexander et al. (2019). In conclusion, miR-433 promotes Agt4a-dependent autophagy.

Finally, because miR-433 is associated with autophagy, which involves the interaction of multiple genes involved in apoptosis, immunity regulation, and metabolism (Kaminskyy and Zhivotovsky, 2014; Poillet-Perez and White, 2019; Tan et al., 2019), we postulated that miR-433 can regulate the expression of these genes. We used the online tool TargetScan 7.2 (www.targetscan.org) to predict possible targets of miRN-433. In addition, Smad9 expression was found to be higher at the mRNA level in the miR-433 group. Smads are the main signal transducers in the TGF-β signaling pathway. Some studies have reported the neuroprotective role of the TGF-β pathway in Alzheimer’s disease (Caraci et al., 2012; Chen et al., 2017). Increased Smad9 expression may be associated with autophagy, but further studies are needed for clarification. In particular, whether miR-433 can induce apoptosis via Smad9 is worth exploring.

Although this study demonstrated that miR-433, an miRNA, is involved in neural development and growth by regulating cell autophagy, there are many questions about the molecular mechanisms involved in the function of miR-433. First, we need to further explore the exact role of miR-433 in neural development using *in vivo* tests. Second, the specific molecular mechanisms of neuronal cells targeted by miR-433 should be further explored. Third, clinical applications of miR-433, especially for neurodegenerative diseases, should be studied.

## Data Availability Statement

The raw data supporting the conclusions of this article will be made available by the authors, without undue reservation, to any qualified researcher.

## Author Contributions

CX and QB contributed equally to this work and should be considered cofirst authors. FW, MC, and JL designed the study. SJ, JZ, and JX prepared the reagents. CW and QM cultured the cells. CX and QB performed the molecular biology experiments. CX, QB, YG, QW, WX, YQ, and YH analyzed and interpreted the data. CX, QB, and JL wrote the document. FW and MC reviewed and revised the document. All authors have reviewed and approved the final version of the manuscript.

## Funding

General Project of Shanghai Natural Science Foundation (18ZR14307000); The Featured Clinical Discipline Project of Shanghai Pudong (PWYst2018-01); Key Discipline Group Construction Project of Shanghai Pudong (PWZxq2017-02).

## Conflict of Interest

The authors declare that the research was conducted in the absence of any commercial or financial relationships that could be construed as a potential conflict of interest.
