# Supplementary figures and images for: miR-433 Inhibits Neuronal Growth and Promotes Autophagy in Mouse Hippocampal HT-22 Cell Line
Source: Front Pharmacol. 2020 Nov 23;11:536913. doi: 10.3389/fphar.2020.536913 (PMC7768889; doi:10.3389/fphar.2020.536913)

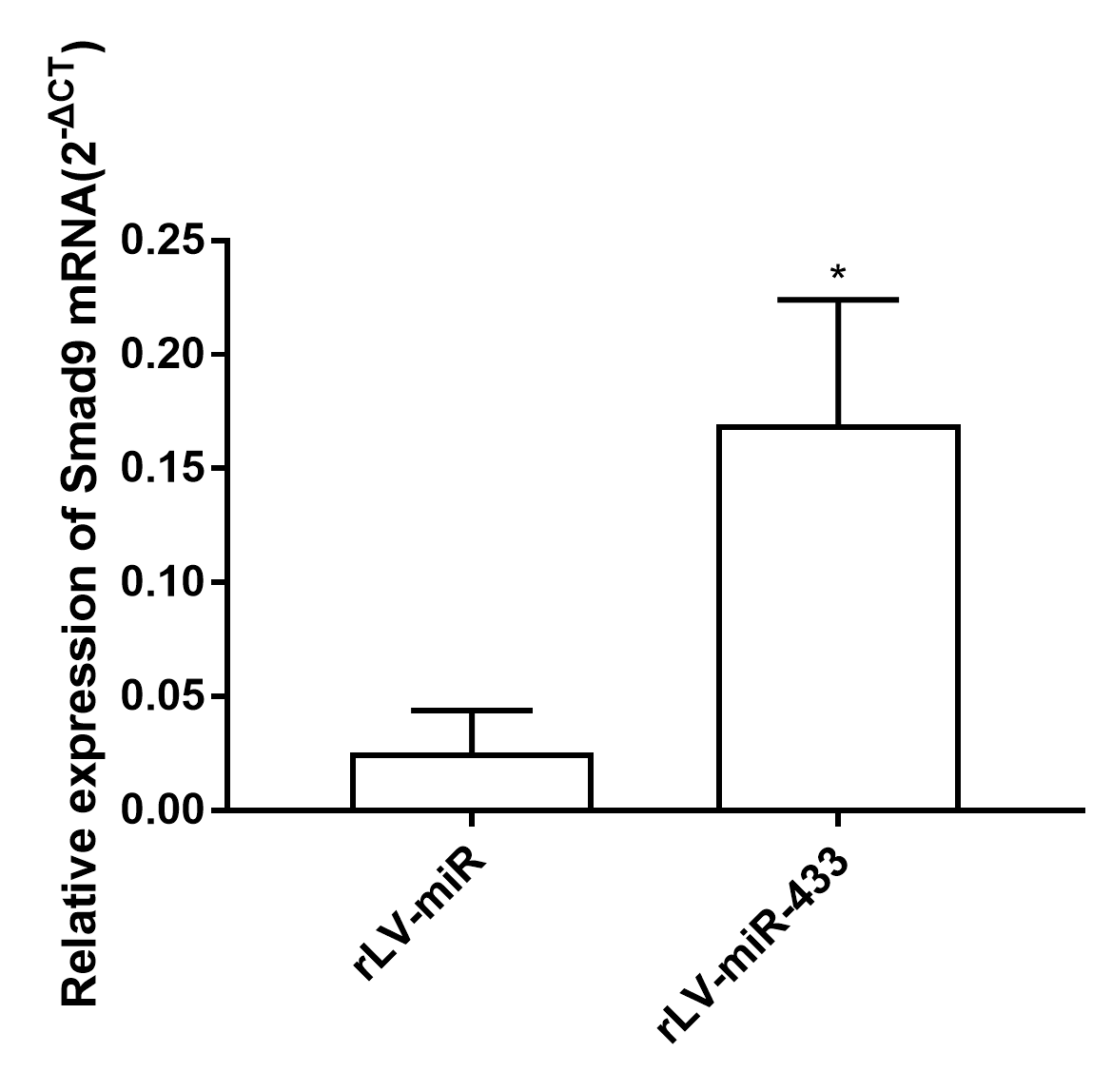

Supplement: Supplementary file 1 [file image1.tif]
